# Case Report: Systemic contact dermatitis caused by benzalkonium chloride in a family (a child and parents)

**DOI:** 10.3389/fped.2025.1531992

**Published:** 2025-06-12

**Authors:** Lu Zhang, Yu-Jie Wang, Yu-Han Ma, Guo-Dong Zhao, Bao-Xiang Zhang

**Affiliations:** ^1^ Department of Dermatology, Yidu Central Hospital of Weifang City, Qingzhou, China; ^2^ School of Clinical Medicine, Shandong Second Medical University, Weifang, China; ^3^ Department of Dermatology, Changle County People’s Hospital, Changle, China

**Keywords:** systemic contact dermatitis (SCD), benzalkonium chloride, pediatric population, allergic reactions, dermatological allergens

## Abstract

**Background:**

Systemic contact dermatitis (SCD) occurs due to re-exposure to sensitizing agents via systemic routes, often triggered by drugs, metals, and food additives. Benzalkonium chloride, a cationic surfactant in different household products, can also induce allergic reactions.

**Case presentation:**

An 11-year-old girl and her parents presented to our outpatient clinic with erythematous, pruritic plaques and scaling. The girl displayed lesions on her anterior chest and popliteal fossae, moreover her parents had similar lesions on their trunks. Notably, she had no previous allergic responses, and furthermore the laboratory results, including blood counts and IgE levels, were normal. Dermoscopic examination revealed a bright-red background with focal branching vasculature and white scales. Histopathology indicated hyperkeratosis, parakeratosis, and dermal inflammation. However, further investigation uncovered that the family had recently started using a laundry detergent containing benzalkonium chloride. What's more, patch testing with dilutions of the detergent was conducted on the girl and her father, both of whom showed positive reactions, which confirming an allergic response. So they were diagnosed as systemic contact dermatitis (SCD). The rash gradually subsided after the detergent was stopped and the anti-allergy treatment was carried out, and there was no recurrence during the 3-month follow-up.

**Discussion:**

Although benzalkonium chloride is mainly regarded as an irritant, it can induce an allergic reaction, particularly in pediatric populations. The case indicates that benzalkonium chloride can trigger SCD even with minimal exposure through household products, and further research is needed to elucidate the underlying immunological mechanisms.

**Conclusion:**

This case emphasizes that benzalkonium chloride can induce SCD even with minimal exposure through household products. Increased awareness of allergens in everyday consumer goods is essential, and further research is needed to elucidate the underlying immunological mechanisms.

## Introduction

Systemic contact dermatitis is a delayed hypersensitivity after systemic re-exposure to a specific hapten to which the individual had been sensitized previously, such as oral ingestion, intramuscular injection, intravenous infusion, halation, or dermal and mucosal absorption (1). Common causative agents include medications, metals, and food additives (2, 3). A well-known subtype of SCD, known as “baboon syndrome,” is characterized by erythema on flexural surfaces induced by certain medications (4). Clinical manifestations of SCD are diverse, often involving recurrence of dermatitis at previously affected skin or patch-test-positive sites. SCD may also present as pruritic vesicular hand eczema, flexural dermatitis, and generalized maculopapular eruptions, among other presentations (4). In some cases, local contact with the skin of a small amount of allergens can also lead to the occurrence of SCD (1).

## Case report

**Figure 1:**
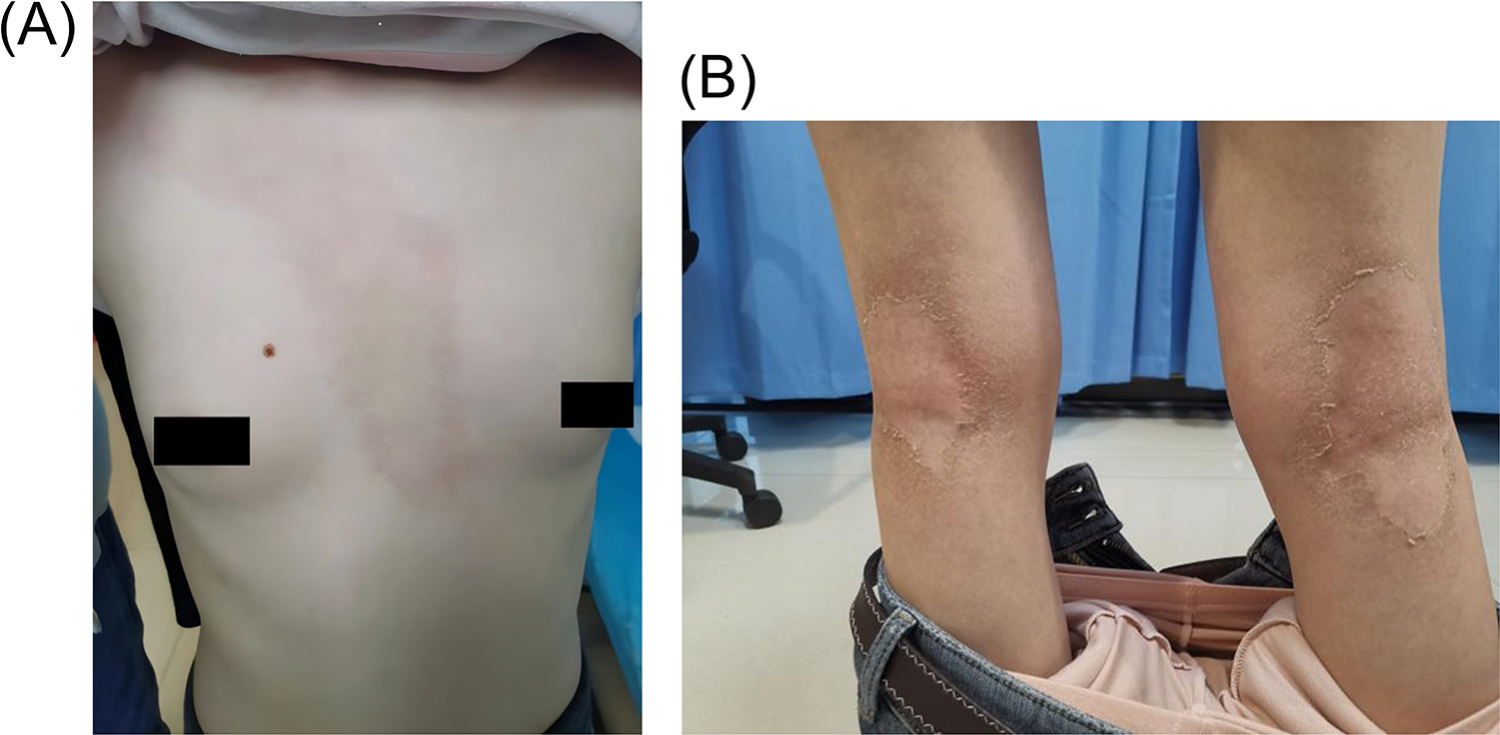
The patient had significant skin lesions visible on the anterior chest **(A)** and bot lower extremes **(B)**. It is mainly characterized by erythema, edema and local changes in skin texture, accompanied by flaking and hyperpigmentation.

**Figure 2:**
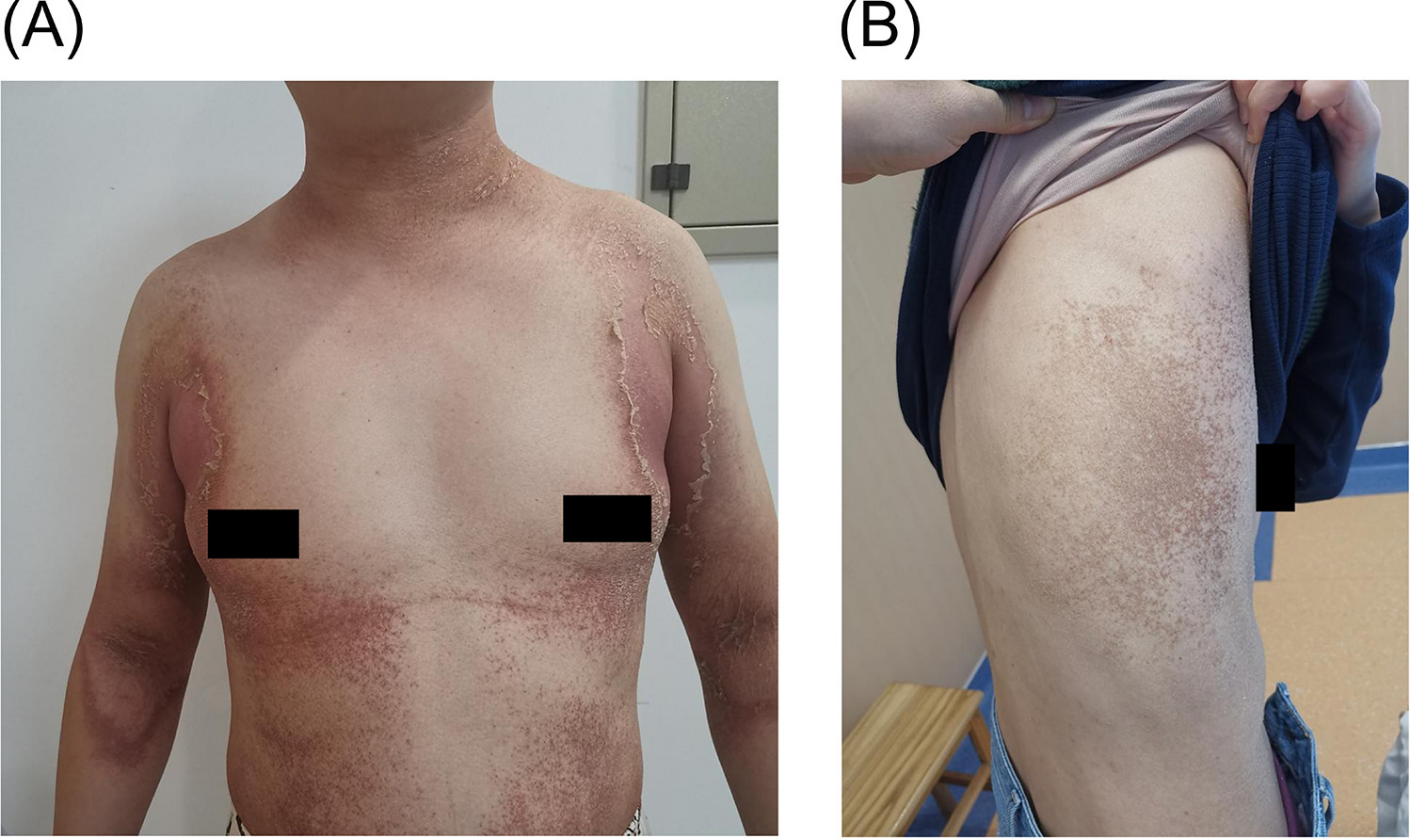
Skin lesions in the patient's family, **(A)** is the parents of the patient, **(B)** is the mother of the patient.

An 11-year-old girl presented with a 5-day history of erythematous plaques, accompanied by scaling and pruritus, localized to the anterior chest and popliteal fossae. Physical examination revealed patchy, faint erythematous plaques with clear borders and visible scaling on the anterior chest and bilateral popliteal fossae (Figure 1). Additionally, the girl's parents also exhibited similar skin lesions on the trunk (Figure 2).

**Figure 3:**
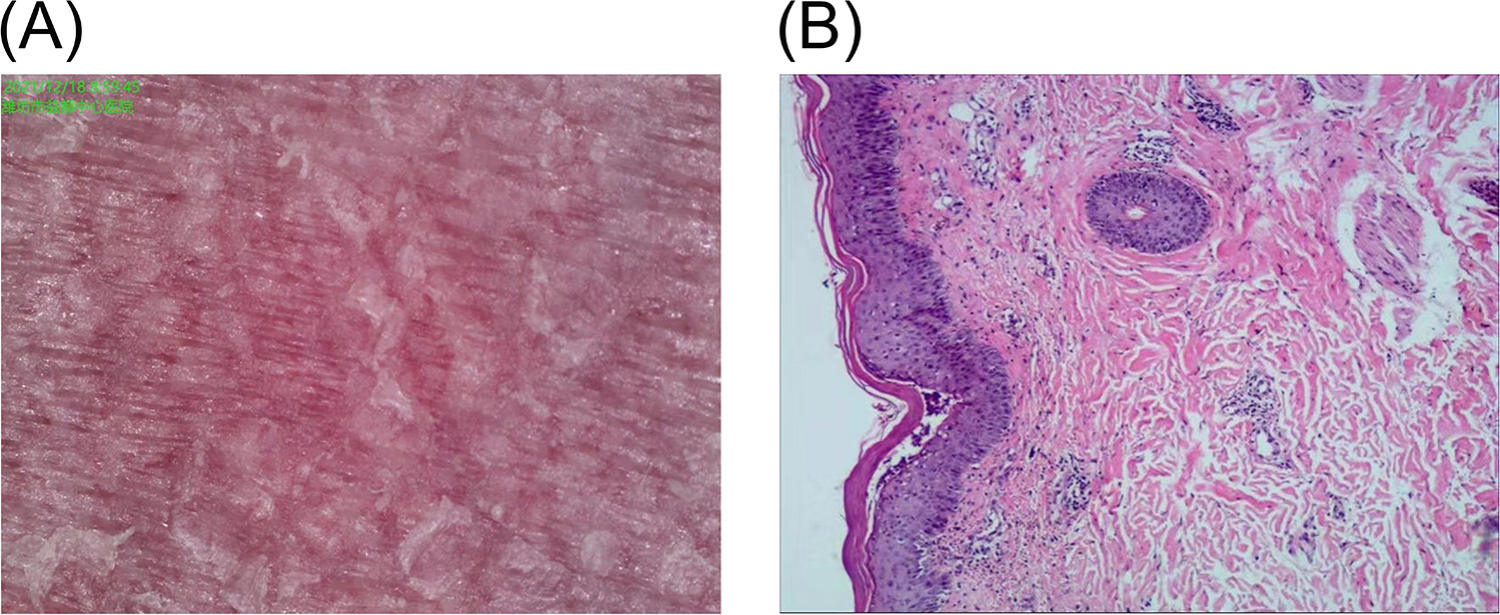
**(A)** Is dermoscopic findings and the **(B)** is histopathologic findings at skin lesions.

The patient reported no previous allergies to medications, foods, or other specific substances. A complete blood count, biochemistry, and total IgE levels were within normal limits. Dermoscopic evaluation revealed a bright-red background, focal branching vasculature, and white scales (Figure 3A). Pathological biopsy specimens were obtained from the skin lesions on the patient's right thigh and the patient's father's lower abdomen, while the mother declined the test. Histopathological examination identified hyperkeratosis, parakeratosis, focal subcorneal microabscesses, koilocytes within the epidermis, erythrocyte extravasation, fibroplasia, hyaline degeneration, and scattered lymphocyte aggregates in the dermis (Figure 3B).

**Figure 4:**
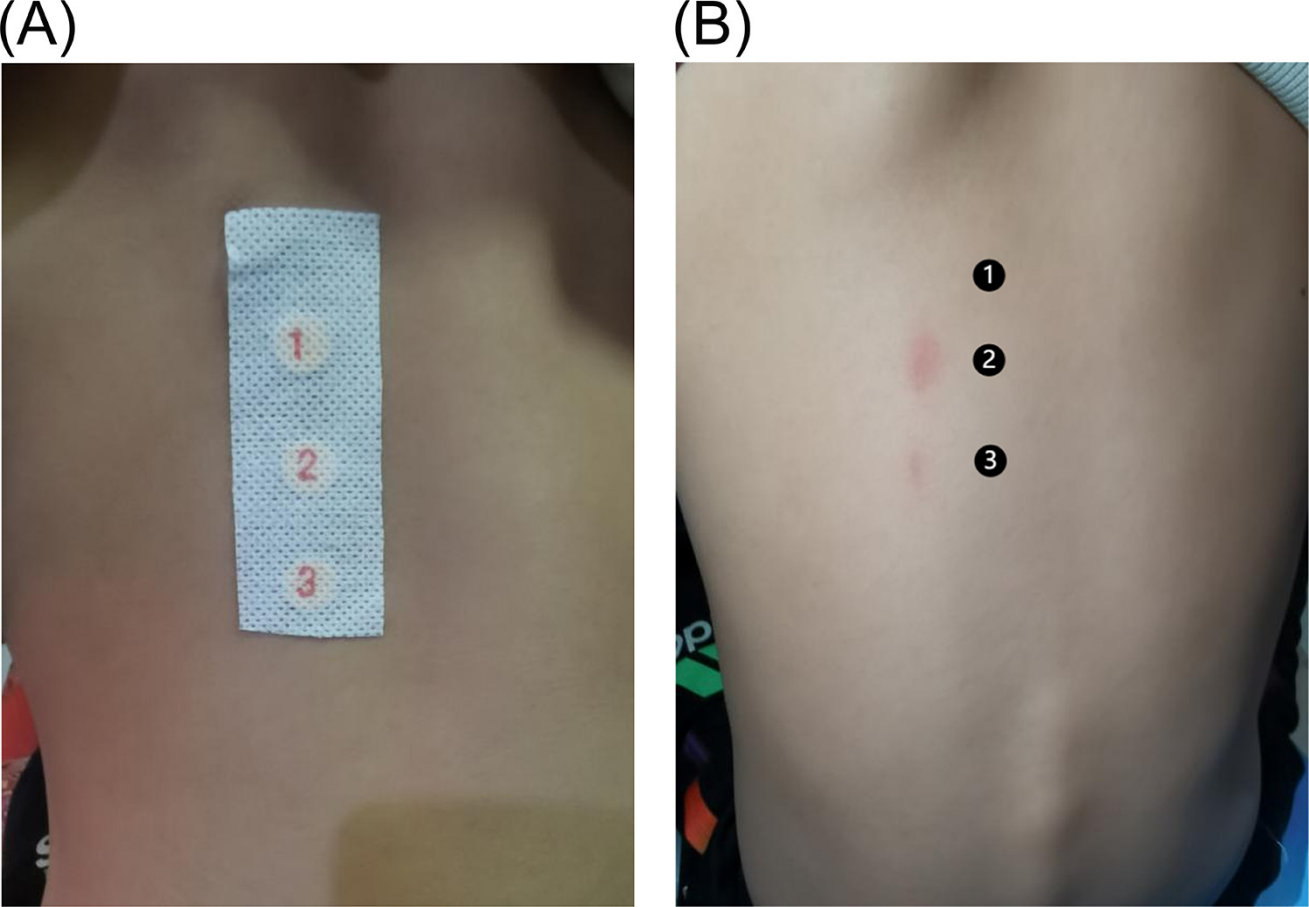
Conduct a patch test on the patient's back. **(A)** Testing material adhesion phase, with ″1″ as a blank control, ″2″ as a detergent dilution 1:100, and ″3″ as a detergent dilution 1:10,000. **(B)** It can be concluded from the results that the patient is allergic to benzalkonium chloride.

Further investigation revealed that all three family members had been exposed to a laundry detergent based on benzalkonium chloride, which they had begun using 6 months prior. After identifying the detergent containing benzalkonium bromide as a suspected allergen, we conducted a patch test on the normal skin of the patient and her father, and both tested positive (Figure 4). However, the mother declined the test.

The pediatric patient presented with erythematous plaques featuring desquamation and pruritus on flexural surfaces, which was consistent with a diagnosis of flexural dermatitis. Upon diagnosis, the detergent was discontinued, and treatment with anti-allergic and moisturizing therapies was initiated for both the patient and her parents. The treatment regimen comprised methylprednisolone, cetirizine, vitamin C, calcium gluconate, and mometasone furoate cream for external use, in addition to basic hydration and moisturizing. Symptoms gradually improved, and a follow-up examination 3 months later confirmed symptom resolution, with no recurrence in either the patient or her parents.

## Discussion

Benzalkonium chloride is a quaternary ammonium compound that exhibits broad-spectrum antimicrobial activity and has prolonged bacteriostatic effects. It is commonly used in cosmetics, disinfectants, and ophthalmic products. Although primarily recognized as an irritant, benzalkonium chloride can, in some cases, exhibit allergenic properties, as evidenced by positive patch test results (5). Hannah et al. reviewed five recent pediatric patch test studies and identified benzalkonium chloride as one of the top allergens responsible for allergic contact dermatitis in children (6).

Further evidence from a retrospective study conducted in France underscores the role of this allergen in pediatric contact dermatitis: from 2010 to 2017, 14 children (with a mean age of 38 months) were diagnosed with contact dermatitis attributed to antiseptics, eight of which were linked to benzalkonium chloride (7). Furthermore, Aaron et al. reported on six pediatric patients who developed granular parakeratosis following exposure to benzalkonium chloride in laundry products. These patients experienced tender, erythematous eruptions in intertriginous areas, with symptoms resolving within 3–4 weeks after discontinuing exposure (8). Another report demonstrated that a child developed an erythema multiforme-like reaction following the use of a potty washed with benzalkonium chloride (9). In our case, prolonged exposure to a detergent containing benzalkonium chloride triggered similar manifestations, including erythematous patches, pruritus, and desquamation, aligning with symptoms documented in prior case reports.

## Limitation

While SCD can occur upon re-exposure to sensitizing haptens, its precise pathophysiology remains incompletely understood. T-cell-mediated hypersensitivity, particularly involving CD8 + CD45RO + CLA + T cells, is believed to play a critical role, with additional contributions from Th1 and Th17 immune pathways (4). Pro-inflammatory cytokines, such as Interleukin-17 (IL-17), IL-22, and Interferon-γ (IFN-γ), have been implicated in allergic contact dermatitis (10). However, their precise role in systemic contact dermatitis has yet to be fully clarified. Additionally, it is known that benzalkonium chloride can compromise the integrity of the skin barrier (11). The potential influence of benzalkonium chloride on antigen presentation and immune activation is not well established. Further investigations, including molecular and genetic studies, are needed to better define these mechanisms and identify potential therapeutic targets. Our diagnosis of benzalkonium chloride-induced SCD was based on patch testing with a dilution of the patient's laundry detergent. However, standardized testing concentrations for benzalkonium chloride allergens remain under debate, and variability in test preparation may affect reproducibility (12, 13). Future studies should aim to include larger cohorts and conduct molecular investigations to deepen our understanding of this condition.

## Conclusion

Benzalkonium chloride possesses irritant and allergenic properties. This case highlights the risk of systemic contact dermatitis from low-dose, prolonged exposure to products containing benzalkonium chloride, such as laundry detergents. Our findings underscore the significance of awareness regarding the allergenic potential of frequently used chemicals and advocate for additional research to elucidate the pathogenesis of SCD.

## Materials and methods

Skin biopsy samples were obtained from the affected areas and immediately fixed in 10% neutral-buffered formalin. The samples were then embedded in paraffin, sectioned at a thickness of 4 μm, and stained with hematoxylin and eosin (H&E) for histopathological examination. Microscopic evaluation was performed by a board-certified dermatopathologist.

## Data Availability

The original contributions presented in the study are included in the article/Supplementary Material, further inquiries can be directed to the corresponding author.
